# Comparative study on the use of specific and heterologous microsatellite primers in the stingless bees *Melipona rufiventris and M. mondury* (Hymenoptera, Apidae)

**DOI:** 10.1590/S1415-47572010005000017

**Published:** 2010-06-01

**Authors:** Denilce Meneses Lopes, Lúcio Antônio de Oliveira Campos, Tânia Maria Fernandes Salomão, Mara Garcia Tavares

**Affiliations:** Departamento de Biologia Geral, Universidade Federal de Viçosa, Viçosa, MGBrazil

**Keywords:** *Melipona*, stingless bees, microsatellite, transferability

## Abstract

Due to their high degree of polymorphism, microsatellites are considered useful tools for studying population genetics. Nevertheless, studies of genetic diversity in stingless bees by means of these primers have revealed a low level of polymorphism, possibly the consequence of the heterologous primers used, since in most cases these were not specifically designed for the species under consideration. Herein we compared the number of polymorphic loci and alleles per locus, as well as observed heterozygosity in *Melipona rufiventris* and *M. mondury* populations, using specific and heterologous primers. The use of specific primers placed in evidence the greater frequency of polymorphic loci and alleles per locus, besides an expressive increase in observed heterozygosity in *M. rufiventris* and *M. mondury*, thereby reinforcing the idea that populational studies should be undertaken by preferably using species-specific microsatellite primers.

## Introduction

Microsatellites or simple sequence repeats (SSR) are regions of the genome made up of short repeat sequences, consisting of one to six nucleotides (Hancock, 1999). Microsatellites have been widely used in studies with various organisms, due to their high degree of polymorphism and co-dominance. One of the limiting stages in the use of these markers is the development of specific primers. However, it has been discovered that the regions flanking microsatellites are very much conserved, and thus primers can be used among species, even among those from different genera (Ferreira and Grattapaglia, 1998). Consequently, the use of heterologous primers has been reported in several studies on bees (Carvalho-Zilse and Kerr, 2006; Francisco *et al.*, 2006; Insuan *et al.*, 2007).

Nevertheless, it is known that by using heterologous primers, the level of information differs among species, generally with a decrease in successful amplification as the genetic distance increases among species (Francisco *et al.*, 2006), thereby inducing a reduction in observed polymorphism. But to what degree does this loss of information lead to mistaken conclusions regarding population genetic structure?

In bees, microsatellite primers were first described for *Apis mellifera* and *Bombus terrestris* (Estoup *et al.*, 1993), and currently there are reports on primers for four stingless bee species: *Melipona bicolor* (Peters *et al.*, 1998), *Scaptotrigona postica* (Paxton *et al.*, 1999), *Trigona carbonaria* (Green *et al.*, 2001) and *M. rufiventris* (Lopes *et al.*, 2009).

Tavares *et al.* (2007) used *M. bicolor* microsatellite primers when assessing genetic diversity in *M. mondury* and *M. rufiventris* populations. They analyzed samples from forest and savanna regions in the state of Minas Gerais, thereby uncovering low genetic diversity in these species, when compared to that found in species analyzed with the use of species-specific primers. However, it is not known whether these estimates were the outcome of using heterologous primers, seeing that some authors reported the presence of null alleles in a like situation (Pépin *et al.*, 1995), or due to the small size of the populations examined (Campos, 1998), with the consequential reduction in genetic diversity.

The objective of the present study was to analyze populations of *M. mondury* and *M. rufiventris* with recently designed specific microsatellite primers (Lopes, 2008) and compare data with those obtained when primers developed for *M. bicolor* were used.

## Material and Methods

###  Biological material

*Melipona mondury* and *M. rufiventris* workers, collected in the state of Minas Gerais, were analyzed (Table 1). Total DNA was extracted, according to the protocol recommended by Waldschmidt *et al.* (1997), by using the adult worker thorax of one bee per colony.

###  Molecular analysis

The bees were analyzed using nine primers specific for *M. mondury* (Mmo08, Mmo10, Mmo11, Mmo15, Mmo19, Mmo20, Mmo21, Mmo22 and Mmo24) (Lopes, 2008), nine primers specific for M. *rufiventris* (Mru03, Mru04, Mru05, Mru06, Mru09, Mru10, Mru11, Mru12 and Mru14) (Lopes *et a*l., 2009) and nine heterologous primers designed for *M. bicolor* (Mbi32, Mbi215, Mbi218, Mbi232, Mbi233, Mbi254, Mbi256, Mbi259 and Mbi278) (Peters *et al.*,1998).

PCR amplifications were carried out in reactions of 10 μL containing 12.5 ng of genomic DNA, 1X Promega *Taq* PCR buffer, 0.5 or 0.25 μM of each forward and reverse primer, 0.1 mM dNTP, 1.5 or 1.0 mM MgCl_2_, and 1U *Taq* DNA polimerase (Promega). The conditions for the PCR were the following: 94 °C (3 min) followed by 40 cycles at 92 °C (30 s), specific pairing temperature for each primer (1 min) and 72 °C (30 s) and a final extension step at 72 °C (5 min). The PCR products were resolved in 8% denaturing polyacrylamide gel and visualized by staining with 0.2% silver nitrate.

The polymorphism level was determined by the number of polymorphic loci (P), the mean number of alleles per locus (A) and observed heterozygosity (H_o_). All these analyses were carried out using the PopGene version 1.32 (Yeh *et al.*, 1999) and TFPGA (Miller, 1997) programs.

## Results and Discussion

The number of polymorphic loci and alleles per locus, as well as heterozygosity in *M. rufiventris* and *M. mondury*, when specific and heterologous primers were used, respectively, appears in Tables 2  and 3.

On comparing the two Tables, it can be seen that all the nine primers designed specifically for these two species, except for one in *M. mondury*, were polymorphic in species-specific amplification, but when *M. bicolor* specific microsatellite primers were used, only three (Mbi218, Mbi232 and Mbi254) and four (Mbi218, Mbi232, Mbi233 and Mbi254) were polymorphic in *M. mondury* and *M. rufiventris*, respectively. It is interesting to note that the same loci were polymorphic in the two species, which can be explained through *M. rufiventris* and *M. mondury* being very close phylogenetically.

It was apparent that the mean number of alleles detected when using species-specific primers was much greater than with heterologous, and that heterozygosity in the two species almost doubled in comparison to that detected when employing primers designed for *M. bicolor*. However, in two heterologous loci (Mbi218 in *M. rufiventris* and Mbi254, in *M. mondury* and *M. rufiventris*), heterozygosity indices were higher than those observed in *M. bicolor.* Furthermore*,* in some loci H_e_ and H_o_ were discrepant. The segregation pattern of these loci was tested using ten workers from each colony and revealed that phenotypes were consistent with that expected for a haplodiploid system. This confirmed that the loci really represented genetic markers and that observed differences were possibly the result of sampling problems.

Low polymorphism levels and high numbers of monomorphic loci have already been detected in several studies of genetic diversity in stingless bees when using heterologous primers (Francisco *et al.*, 2006; Borges, 2007; Silva, 2007; Tavares *et al.*, 2007). For example, observed heterozygosity detected in populations of *Partamona helleri*, *Plebeia remota* (Francisco *et al.*, 2006) and *M. mondury* (Tavares *et al.*, 2007) was 0.11, 0.24 and 0.12, respectively. This was much lower than what was detected in *M. bicolor* (H_omean_ = 0.40), the species for which the primers were originally designed.

This is mainly explained by the presence of null alleles that are not amplified because of mutations in the primer pairing sequence (Callen *et al.*, 1993). In general, the number of alleles and genetic diversity are greater in species for which microsatellite primers were originally designed, and successful transferability is usually inversely proportional to mutual genetic distance (Primmer and Merilä, 2000). Carvalho-Zilse and Kerr (2006) observed this in *M. scutellaris,* where the success of transferability was greater with the use of primers specifically designed for *M. bicolor*, a species of the same genus, than with those for *Apis mellifera*. Similarly, Lopes (2008) observed that the transferability of primers designed for *M. rufiventris* and *M. mondury* was higher in other *Melipona* species than in *Partamonahelleri.*

Another important mechanism to be considered when using heterologous primers is preferential amplification of one of the alleles during PCR, thereby possibly hindering detection of individual heterozygotes. In this case, the enzyme used in the process would be more active in amplifying the smaller sized allele, thereby generating an increase in the concentration of this in detriment to the larger one (Wattier *et al.*, 1998).

From our data, it can be seen that, although heterologous primers can be successfully used in studies of phylogenetically close species, the results should be carefully analyzed, as the number of alleles and heterozygosity revealed in each case can vary, depending on various factors. Thus, the use of specific primers should be preferred in comparison to the heterologous. Nevertheless, it was shown that genetic diversity in the two *Melipona* species analyzed was low when compared to other bee species, even when using specific microsatellite primers (Peters *et al.*, 1998; Paxton *et al.*, 2003; Carvalho-Zilse and Kerr, 2006; Souza *et al.*, 2007). For example, observed heterozygosity in *M. scutellaris* (H_o_ = 0.315 - Carvalho-Zilse and Kerr, 2006), was found to be higher than in *M. rufiventris* (H_o_ = 0.16) and *M.* mondury (H_o_ = 0.22) (present study). The low number of colonies found in several localities, as a consequence of habitat destruction and fragmentation, as well as predatory honey collecting, may be contributing to the reduction in genetic variability. In fact, in Minas Gerais, *M. rufiventris* and *M.mondury* consist of small local populations and these species have being so depleted, they are now considered endangered species (Campos, 1998).

## Figures and Tables

**Table 1:** Locality and number of the colonies of *Melipona rufiventris* and *M. mondury* analyzed.

Species	Locality	Number of colonies
	Guimarânia	9
	Patos de Minas	4
	Patrocínio	2
	Arcos	2
*M. rufiventris*	Uberaba	2
	Córrego Danta	3
	Pequi	1
	Formiga	2

	Total	25

	Coluna	1
	Itamarandiba	3
	Resende Costa	6
*M. mondury*	Rio Vermelho	5
	Pote	4
	Diogo Vasconcelos	3
	Marliéria	1

	Total	23

**Table 2:** Diversity parameters for nine microsatellite primers designed specifically for *Melipona rufiventris* and *M. mondury*.

*M. rufiventris*					*M. mondury*			
Locus	A	H_o_	H_e_		Locus	A	H_o_	H_e_
Mru03	3	0.43	0.62		Mmo08	1	0.00	0.00
Mru04	5	0.32	0.43		Mmo10	2	0.00	0.23
Mru05	2	0.00	0.15		Mmo11	2	0.17	0.16
Mru06	4	0.13	0.16		Mmo15	2	0.43	0.48
Mru09	4	0.08	0.49		Mmo19	8	0.60	0.80
Mru10	3	0.26	0.57		Mmo20	2	0.00	0.23
Mru11	4	0.21	0.54		Mmo21	5	0.27	0.64
Mru12	3	0.00	0.63		Mmo22	6	0.45	0.81
Mru14	2	0.00	0.32		Mmo24	2	0.09	0.08

Mean	3.3	0.16	0.43		Mean	3.3	0.22	0.38

A: number of alleles. H_o_ and H_e_: observed and expected Nei heterozygosity, respectively.

**Table 3:** Diversity parameters estimated for *Melipona rufiventris* and *M. mondury*, using nine microsatellite primers designed specifically for *M. bicolor*.

Locus	*M. rufiventris*			*M. mondury*			M. bicolor*	
	A	H_o_		A	H_o_		A	H_o_
Mbi32	1	0.00		1	0.00		4	0.63
Mbi215	1	0.00		1	0.00		3	0.50
Mbi218	4	0.28		4	0.09		3	0.12
Mbi232	2	0.00		3	0.09		4	0.88
Mbi233	1	0.00		5	0.43		6	0.88
Mbi254	3	0.48		4	0.57		3	0.38
Mbi256	1	0.00		1	0.00		4	0.50
Mbi259	1	0.00		1	0.00		2	0.12
Mbi278	1	0.00		1	0.00		5	0.86

Mean	1.67	0.09		2.33	0.13		3.78	0.54

*Peters *et al.* (1998).
